# WWP2 in immune surveillance and antigen presentation: context-dependent roles in B cells, T cells, and tumor immune escape

**DOI:** 10.3389/fimmu.2026.1966504

**Published:** 2026-09-15

**Authors:** Wanchen Lu, Yinan Zhu, Haiyan Xi, Xuyong Lin

**Affiliations:** Department of Pathology, The First Hospital of China Medical University, Shenyang, Liaoning, China

**Keywords:** B cell tolerance, HECT E3 ubiquitin ligase, immune surveillance, MHC-I antigen presentation, T cell regulation, tumor immune escape, ubiquitination, WWP2

## Abstract

WWP2 is a NEDD4-family HECT E3 ubiquitin ligase with emerging relevance to immune regulation and cancer immunity. Although WWP2 has been widely studied in tumor growth, fibrosis, and cell signaling, its immune functions are less consistently integrated into current models of cancer immune surveillance. This review summarizes the structural and regulatory features of WWP2 and examines how its substrate- and adaptor-dependent activities influence B cell tolerance, helper T cell responses, innate immune signaling, and tumor immune escape. Particular attention is given to the SUSD6/TMEM127/WWP2 axis, which promotes MHC-I ubiquitination and lysosomal degradation, thereby reducing cell-surface antigen presentation and potentially weakening cytotoxic T cell recognition. By separating immune, tumor-intrinsic, and context-specific mechanisms, we highlight WWP2 as a conditional regulator of antigen presentation rather than a uniformly oncogenic ligase. A more precise understanding of WWP2-dependent immune pathways may support biomarker development and guide therapeutic strategies that restore antigen presentation or selectively disrupt pathogenic adaptor-ligase interactions.

## 1 Introduction

Ubiquitination is a central post-translational modification that controls protein stability, subcellular localization, and signal transduction. Its specificity is largely determined by E3 ubiquitin ligases, which select substrates and define whether ubiquitin modification leads to proteasomal degradation, altered trafficking, or remodeling of signaling complexes (1–3). Because ubiquitination also regulates immune-defence signaling, E3 ligases are increasingly viewed as pathway-specific immune regulators rather than housekeeping enzymes alone (4).

HECT-type E3 ligases are distinguished by catalytic transfer of ubiquitin through an active-site cysteine before substrate modification (5, 6). WWP2, a member of the NEDD4 family, contains an N-terminal C2 domain, four WW domains, and a C-terminal HECT domain, giving the protein a modular architecture for membrane association and substrate recognition (7–9). The HECT domain completes ubiquitin transfer and is regulated by catalytic and conformational features that have been characterized biochemically (10, 11). Isoform diversity and intramolecular regulation further expand the range of WWP2-dependent outcomes across cell types (12, 13).

Accumulating studies place WWP2 at the intersection of tissue remodeling, tumor biology, and immune regulation. In non-malignant disease models, WWP2 has been linked to SMAD2-dependent fibrosis and pro-fibrogenic monocyte infiltration (14, 15). It has also been implicated in PARP1-associated cardiac remodeling and vascular injury responses (16, 17). In cancer models, WWP2 can regulate PTEN-AKT signaling and Hippo-YAP activity (18–20), as well as apoptosis-associated programs and Notch signaling (21, 22). These findings support a substrate- and context-dependent view of WWP2 rather than a single uniformly oncogenic function.

The immunological relevance of WWP2 is increasingly recognized. WWP2 limits TLR3 signaling through TRIF degradation (23). It cooperates with Itch in helper T cell regulation and autoimmune neuroinflammation (24, 25). It has also been implicated in B cell tolerance and macrophage stress responses (26, 27). Most directly for tumor immunity, the SUSD6/TMEM127/WWP2 axis promotes MHC-I ubiquitination and lysosomal degradation (28). This mechanism links WWP2 to crucial roles in immunoediting and the cancer-immunity cycle (29–31), MHC class I recovery and antigen presentation (32, 33), MHC-I loss and downregulation (34, 35), antigen-presentation control and immune evasion (36–38), and the NLRC5-dependent regulation of MHC class I expression (39). Broader reviews position E3 ligases as cancer-immunity regulators (40). This review therefore examines WWP2 as a context-dependent regulator of immune surveillance, rather than as a simple tumor-promoting ligase.

## 2 Molecular organization and regulatory logic of WWP2

WWP2 contains an N-terminal C2 domain, four WW domains, and a C-terminal HECT domain, as summarized in **Figure 1** (8, 9). This arrangement gives the ligase three distinct capacities: subcellular positioning, substrate or adaptor recognition, and catalytic ubiquitin transfer. The C2 domain contributes to membrane association and trafficking-related localization. The WW domains bind proline-rich recognition motifs in substrates or adaptor proteins. The HECT domain accepts ubiquitin from an E2 enzyme through its catalytic cysteine before transferring ubiquitin to the target protein (8–11).

**Figure 1:**
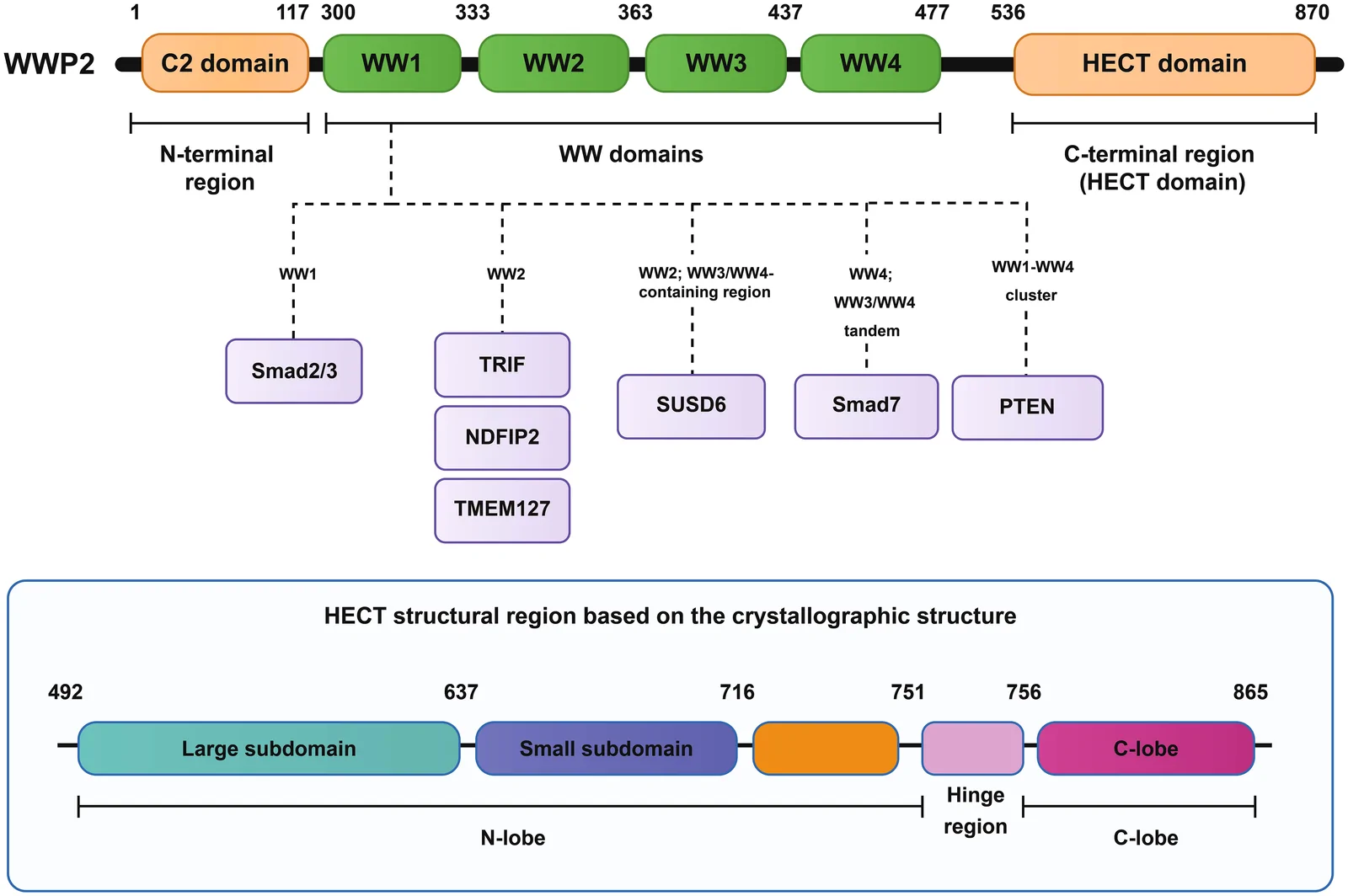
Domain organization of WWP2, representative interaction partners, and structural organization of its HECT catalytic region. Full-length WWP2 contains an N-terminal C2 domain, four WW domains (WW1–WW4), and a C-terminal HECT domain. Representative interaction partners are shown together with their reported WWP2-binding regions. Smad2/3 associate with the WW1-containing region, whereas TRIF, NDFIP2, and TMEM127 are associated with WW2. SUSD6 interacts with WW2 and with regions containing WW3/WW4, while Smad7 interacts with WW4 and the WW3–WW4 tandem region. PTEN binding is represented by the broader WW1–WW4 cluster. The lower inset illustrates the structural organization of the WWP2 HECT region, including the N-lobe, hinge region, and C-lobe. Where single-WW-domain resolution is unavailable, a broader WW-domain region is indicated based on the available experimental evidence.

The WW domains are particularly important for specificity. Individual WWP2 WW domains differ in binding affinity and partner preference, so the same ligase can engage different proteins in different cellular environments (9). This helps explain why WWP2 can participate in apparently unrelated pathways such as PTEN regulation, Smad signaling, Notch control, and MHC-I trafficking (12, 19, 22, 28). The key point is that WWP2 does not act as a single-purpose oncogene. Its effect depends on the recruited substrate and the adaptor complex that brings the substrate to WWP2.

The HECT domain provides the catalytic step of the reaction. Its N-lobe interacts with ubiquitin-loaded E2 enzymes, whereas its C-lobe contains the catalytic cysteine (8, 10, 11). Ubiquitin transfer requires conformational movement between the two lobes. This feature is relevant to immune and cancer biology because WWP2 can change the abundance of a target protein, alter receptor trafficking, or terminate a signaling complex. The biological outcome is therefore determined not only by whether WWP2 is expressed, but also by which substrate is accessible at a given time.

WWP2 is further diversified by alternative transcript usage and isoform-specific domain composition. The best-characterized forms include full-length WWP2 (WWP2-FL), the N-terminal WWP2-N isoform, and the C-terminal WWP2-C isoform (12, 13). WWP2-FL contains the complete C2–WW–HECT architecture, whereas WWP2-N retains the N-terminal portion and WW1 domain but lacks the C-terminal HECT catalytic domain. By contrast, WWP2-C contains the C-terminal region, including WW4 and the HECT domain (12). These structural differences translate into distinct substrate preferences: WWP2-N preferentially interacts with Smad2/3, whereas WWP2-C preferentially interacts with Smad7; full-length WWP2 can engage both groups of Smads and shows a strong functional preference for Smad7 turnover (12). WWP2-N can also interact with WWP2-FL and modulate its activity, illustrating that truncated isoforms may regulate WWP2 signaling even when they do not possess an autonomous catalytic HECT domain (12, 13).

Evidence also suggests that WWP2 isoform abundance can vary across tumor contexts. Isoform-specific analyses of human cancer cDNA panels have reported reduced WWP2-N expression in advanced melanoma, increased WWP2-N expression in stage II/III prostate cancer, and isolated increases in WWP2-FL or WWP2-C in early-stage breast cancer (13). These observations indicate that gene-level WWP2 abundance may conceal biologically distinct isoform patterns. In contrast, isoform-resolved expression and function of WWP2 in defined human immune-cell populations remain insufficiently characterized. Most studies of WWP2 in T cells, B cells, macrophages, or innate immune signaling have examined total WWP2 expression, genetic deletion, or overall protein activity rather than systematically resolving individual isoforms (23–27). Consequently, a direct tumor-cell-versus-immune-cell atlas of WWP2 isoforms is currently lacking. This limitation is particularly relevant when interpreting clinical transcriptomic datasets, because published analyses that use gene-level WWP2 expression cannot establish which isoform, adaptor complex, or substrate-specific activity underlies an observed clinical association. Isoform-aware transcriptomic or proteomic approaches will therefore be needed to determine whether specific WWP2 variants carry distinct prognostic or immunological significance.

This modular architecture creates several possible intervention points. A strategy could inhibit HECT catalytic activity, prevent a WW-domain interaction, or block a disease-specific adaptor (8–11). Another strategy is to alter WWP2 abundance pharmacologically, an approach conceptually aligned with targeted protein degradation (41, 42). These approaches are not equivalent. Catalytic inhibition would affect many WWP2 functions, whereas adaptor- or substrate-selective approaches may be more suitable when a single pathogenic pathway has been defined.

Thus, the structural biology of WWP2 should be used to guide a substrate-centered view of its function. In cancer, this approach prevents the common mistake of treating all WWP2-associated pathways as evidence for one universal role, a point that is especially relevant to immunoediting and the cancer-immunity cycle (29–31). It instead asks which WWP2-dependent event is active in a particular tumor and whether that event affects growth, metastasis, antigen presentation, or the surrounding immune microenvironment.

## 3 WWP2 as a potential target for immunotherapy

WWP2 first matters for an immunology audience because it shapes B cell tolerance, helper T cell balance, and myeloid stress responses (23–27). It also influences proliferation, survival, migration, and therapy response in selected tumor systems (18–22). These effects are evident in selected experimental systems, although they arise through different substrates in different tumor types. PTEN-AKT signaling, Hippo-YAP signaling, Notch signaling, and apoptosis-associated pathways should therefore be treated as separate mechanisms rather than collapsed into a generic statement that WWP2 promotes all aspects of cancer progression. For the broader ubiquitin field, immune-defence signaling and HECT-family biology already show strong context dependence (4–6).

### 3.1 WWP2, PTEN, and growth signaling

One of the best-known cancer-related mechanisms involves PTEN. PTEN restrains PI3K-AKT signaling and thereby limits growth and survival signals. In tumor models in which WWP2 promotes PTEN ubiquitination and degradation, reduced PTEN protein abundance can increase AKT activity and support proliferation (18, 19). This mechanism has been reported in endometrial cancer and other contexts, but its importance should be assessed together with PTEN genetic status and alternative PTEN-regulatory mechanisms in each tumor type.

The practical implication is not simply that high WWP2 expression predicts growth. Rather, a PTEN-dependent WWP2 phenotype is most plausible when tumor cells retain PTEN that can be regulated at the protein level (18, 19). This distinction matters for therapeutic targeting because a WWP2 inhibitor is unlikely to restore a PTEN pathway that has already been eliminated by genomic deletion or inactivating mutation.

### 3.2 WWP2, hippo signaling, and invasive behavior

WWP2 has also been linked to the Hippo pathway. In gastric cancer models, WWP2-mediated ubiquitination of LATS1 has been associated with reduced Hippo pathway restraint and increased YAP activity (20). Because YAP can induce genes involved in proliferation, migration, and matrix remodeling, this pathway provides a coherent explanation for how WWP2 may promote aggressive behavior in some tumors.

The migration phenotype should nevertheless be described with caution. Invasion is not a single pathway outcome; it reflects cell adhesion, cytoskeletal remodeling, extracellular matrix degradation, epithelial-mesenchymal plasticity, and stromal interactions. WWP2 may contribute to these processes through LATS1-YAP signaling or other substrates, but the responsible mechanism needs to be established in each tumor model. The available evidence supports a context-dependent role in invasive behavior rather than a universal metastasis program.

### 3.3 WWP2 and apoptosis resistance

Several cancer studies associate WWP2 with apoptosis resistance. In hepatocellular carcinoma models, WWP2 silencing has been reported to increase pro-apoptotic proteins, including caspase family members and Bax, while reducing Bcl-2 (21). These observations are consistent with a survival-promoting role. However, changes in apoptosis markers do not by themselves identify the direct WWP2 substrate. Future work should distinguish direct ubiquitin targets from secondary changes caused by altered growth signaling or cellular stress.

Together, the tumor-intrinsic literature supports WWP2 as a regulator of selected oncogenic circuits. The strongest interpretation is not that WWP2 is uniformly oncogenic, but that it can favor tumor growth or dissemination when it is coupled to susceptible substrates such as PTEN or LATS1. This substrate dependence becomes even more important when WWP2 is considered as an immunotherapy target. Selected tumor-intrinsic WWP2-associated pathways and representative findings across cancer models are summarized in **Figure 2**, **Table 1**, respectively.

**Figure 2:**
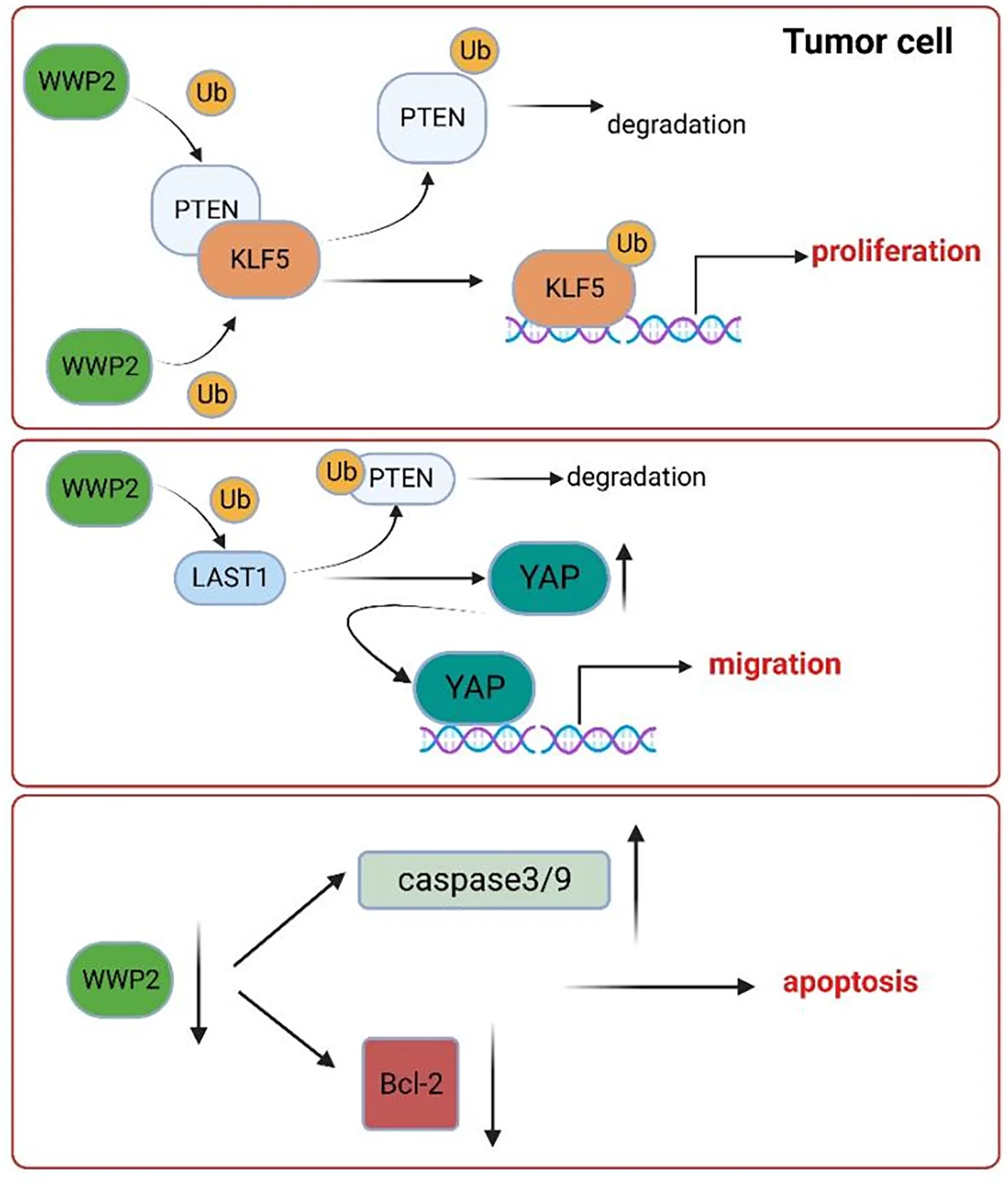
Schematic representation of selected tumor-intrinsic signaling pathways associated with WWP2. WWP2-mediated ubiquitination of signaling proteins can alter their stability and downstream tumor-cell phenotypes in a substrate-dependent manner. In PTEN-associated signaling, WWP2-dependent ubiquitination and degradation of PTEN can relieve restraint on growth-promoting pathways and contribute to tumor-cell proliferation. In the Hippo pathway, WWP2-mediated ubiquitination and degradation of LATS1 reduces Hippo pathway restraint, thereby increasing YAP activity and promoting migration-associated transcriptional programs. WWP2 has also been associated with regulation of apoptosis-related proteins; reduced WWP2 activity has been reported to increase caspase-associated apoptotic signaling and reduce the anti-apoptotic protein Bcl-2 in selected tumor models. Arrows indicate the direction of the reported regulatory relationships, and “Ub” denotes ubiquitin. PTEN, phosphatase and tensin homolog; LATS1, large tumor suppressor kinase 1; YAP, Yes-associated protein; Bcl-2, B-cell lymphoma 2.

**Table 1:** Representative tumor-intrinsic functions of WWP2 reported across cancer models.

Tumor type	Reported WWP2-associated phenotype	Mechanistic interpretation	Reference
Endometrial cancer	Increased proliferation and survival	WWP2-associated PTEN ubiquitination may increase PI3K-AKT signaling when PTEN remains available for protein-level regulation.	(19)
Colorectal cancer	Altered growth signaling	WWP2 expression has been linked to changes in Hippo pathway phosphorylation and YAP-dependent proliferative outputs; pathway dependence should be validated by model.	(60)
Lung cancer	Enhanced invasion-associated behavior	Reported links to extracellular matrix remodeling and invasive signaling suggest a context-dependent contribution to migration and metastasis.	(60)
Gastric cancer	Enhanced migration and invasion	WWP2-mediated LATS1 degradation can weaken Hippo restraint and increase YAP target gene expression, including CTGF and CYR61.	(20)
Hepatocellular carcinoma	Apoptosis resistance	WWP2 silencing has been associated with increased caspase/Bax expression and reduced Bcl-2, although the direct ubiquitin target remains to be established.	(21)

## 4 WWP2 at the interface of tumor biology and immune surveillance

The immunological relevance of WWP2 should be judged by evidence strength. Direct immune mechanisms are those in which WWP2 has been shown to regulate TLR3/TRIF signaling (23), helper T cell differentiation (24, 25), B cell tolerance (26), macrophage stress responses (27), or MHC-I antigen-presentation machinery (28). Indirect immune relevance refers to tumor-cell pathways that may influence the microenvironment, such as PI3K-AKT, YAP, or TGF-beta-related programs (18–20, 43, 44). This distinction is essential. It prevents a review from presenting plausible tumor microenvironment hypotheses as if they were already established WWP2 immune mechanisms. These distinct levels of evidence and their implications for tumor immunity are summarized in **Table 2**.

**Table 2:** Direct evidence and proposed extensions for WWP2 in tumor immune escape.

Evidence category	WWP2-associated mechanism	Interpretation for tumor immunity	Reference
Direct innate immune evidence	TRIF ubiquitination and degradation downstream of TLR3	WWP2 limits TLR3-dependent inflammatory and antiviral signaling; consequences may differ between inflammation and antitumor immunity.	(23)
Direct adaptive immune evidence	Cooperation with Itch in TCR-linked helper T cell regulation	Supports context-specific regulation of helper T cell differentiation, not a general claim of direct T cell dysfunction in tumors.	(24, 25)
Direct cancer-immunity evidence	SUSD6/TMEM127-dependent recruitment of WWP2 to MHC-I	Promotes MHC-I ubiquitination and lysosomal degradation, reducing antigen presentation to CD8+ T cells.	(28)
Direct evidence outside cancer	Regulation of fibrosis-associated monocyte activity and remodeling	Provides a rationale to examine WWP2 in immune-stromal crosstalk, but does not prove a tumor microenvironment mechanism.	(14, 15)
Proposed tumor-microenvironment extensions	Possible links to TGF-beta, IL-10, macrophages, MDSCs, or Tregs	Biologically plausible but not established as direct WWP2 mechanisms; requires direct tumor-model evidence.	(43–47)

### 4.1 Tumor microenvironment: plausible links, limited direct evidence

WWP2 has documented roles in fibrosis-associated signaling and pro-fibrotic monocyte activity in the heart (14, 15). These findings are relevant to cancer because fibrotic stroma and myeloid cells can restrict T cell infiltration and support immune exclusion (45–47). They provide a rationale for investigating WWP2 in tumor-associated macrophages, monocytes, fibroblasts, and stromal compartments. However, direct evidence that WWP2 controls MDSC recruitment, macrophage polarization, regulatory T cells, or immune-excluded architecture in tumors remains limited.

### 4.2 Innate and adaptive immune signaling

WWP2 has direct roles in defined immune pathways. In innate immunity, WWP2 targets the TLR3 adaptor TRIF for ubiquitination and degradation (23). This limits downstream antiviral and inflammatory signaling. In adaptive immunity, WWP2 cooperates with the E3 ligase Itch in TCR-dependent helper T cell regulation (24, 25). These studies show that WWP2 can tune immune activation, but they do not support a broad claim that WWP2 is uniformly immunosuppressive.

The TRIF mechanism illustrates the importance of context. Limiting TLR3 signaling may protect tissues from prolonged inflammation (23). In a tumor or antigen-presenting-cell context, however, excessive TRIF degradation could reduce interferon-associated immune activation. The same molecular event may therefore be beneficial or harmful depending on whether the clinical goal is to restrain inflammation or improve antitumor immunity.

The Itch-WWP2 pathway also requires careful interpretation. Available studies support a role in TCR signaling and helper T cell differentiation, including restraint of excessive TH2 responses and regulation of helper T cell plasticity in autoimmune settings (24, 25). These data support a context-specific role in helper T cell regulation, not a generalized model in which WWP2 directly drives terminal T cell dysfunction in human tumors. More recent work extends this immune scope to B cell tolerance, where LAPTM5 uses WWP2 to tune the PTEN-AKT axis (26), and to macrophage host response, where WWP2 contributes to TFEB-dependent stress control during infection (27).

For cancer immunotherapy, these immune-cell data create a safety constraint. Systemic WWP2 inhibition might improve one tumor-cell phenotype while changing TLR3 signaling or helper T cell balance elsewhere (23–25). A successful strategy will therefore need to identify the pathologic WWP2 complex, the relevant cell compartment, and the immune outcome that is intended to change.

### 4.3 MHC-I degradation: the most direct WWP2-dependent immune-evasion mechanism

The clearest cancer-immunity mechanism involving WWP2 is the regulation of MHC-I antigen presentation. MHC-I biology has a defined architecture: HLA heavy chain, β2M, and peptide loading through TAP1/TAP2 and tapasin (32, 33). Its transcriptional control is shaped by interferon signaling, NF-κB, IRF1/2, and NLRC5 (34–39). In acute myeloid leukemia and reported solid tumor systems, SUSD6 and TMEM127 recruit WWP2 to MHC-I molecules. WWP2 then promotes their ubiquitination and lysosomal degradation, reducing cell-surface MHC-I abundance and weakening antigen presentation (28).

This SUSD6/TMEM127/WWP2 axis should be the central immune-evasion mechanism in a WWP2-focused cancer review. It directly connects an E3 ligase to impaired antigen presentation (28, 34, 35). It also fits broader studies on MHC-I expression and immune evasion in cancer (36–39).

Other tumor microenvironment links, including TGF-beta-associated immune exclusion, IL-10 signaling, and myeloid suppression, should be positioned as research directions rather than established WWP2 mechanisms (43–47). These pathways matter because checkpoint resistance, T cell dysfunction, and myeloid diversity all shape the immune context of tumors (48–51). The distinction does not weaken the manuscript. It makes the review more credible by showing where evidence is decisive, where it is suggestive, and where direct experiments are still required.

### 4.4 Context dependence and sources of discordant observations

Although the SUSD6/TMEM127/WWP2 axis provides a compelling mechanism for post-translational MHC-I suppression, the magnitude of WWP2 dependence is not uniform across experimental systems. In the original study defining this axis, WWP2 ablation increased surface HLA expression less strongly than loss of SUSD6 or TMEM127, and the E3 ligase STUB1 was also found to associate with SUSD6 or TMEM127, suggesting partial functional redundancy among E3 ligases involved in MHC-I turnover (28). More recently, under hypoxic conditions in HT29 colorectal cancer cells, SUSD6 depletion restored surface MHC-I, whereas TMEM127 depletion had little effect; inhibition of STUB1 also increased surface MHC-I. These findings support the existence of alternative, microenvironment-dependent routes of MHC-I degradation and indicate that the SUSD6/TMEM127/WWP2 complex should not be assumed to function as an invariant module in every tumor context (52).

Several factors may account for such apparently discordant observations. First, WWP2 activity depends on the availability of substrate-recruiting and activating adaptors rather than on WWP2 abundance alone. Recent mechanistic work showed that SUSD6 and TMEM127 can function as a co-disinhibitory pair for WWP2 and engage its WW domains, whereas other adaptor systems can activate WWP2 through distinct interaction modes (53). Second, functional redundancy with other E3 ligases, including STUB1 in the regulation of MHC-I, can reduce the phenotypic effect of WWP2 loss (28, 52). Third, cell type, tumor genotype, microenvironmental conditions, and WWP2 isoform composition can alter substrate accessibility and pathway output. These variables also complicate interpretation of clinical transcriptomic studies: total WWP2 expression does not necessarily report the activity of a specific WWP2–adaptor–substrate complex. Accordingly, associations between WWP2 expression, immune phenotypes, or response to immune-checkpoint blockade should be regarded as context dependent and hypothesis-generating unless the relevant adaptor, substrate, isoform, and cellular compartment are also defined.

## 5 A substrate-centered view of the WWP2 target network

The target network of WWP2 is broad, but its biological meaning becomes clearer when targets are grouped by the phenotype they control. Some substrates are linked to cancer-cell behavior, others to cardiac or vascular stress, and others to antigen presentation or immune signaling. This framework avoids treating WWP2 as a collection of unrelated facts and instead asks how specific substrate-adaptor pairs create disease-relevant outcomes.

### 5.1 Disease-associated WWP2 targets

#### 5.1.1 PARP1 in cardiac remodeling

In cardiac remodeling models, WWP2 has been linked to ubiquitin-dependent regulation of PARP1 (16). This pathway affects stress responses, energy balance, hypertrophic remodeling, and fibrosis. The WWP2-PARP1 axis illustrates that WWP2 can have protective or adaptive functions outside cancer. It also explains why a systemic anti-WWP2 strategy should be evaluated for cardiovascular toxicity rather than assumed to be selectively antitumor.

#### 5.1.2 MHC-I in tumor immune evasion

MHC-I is the most important immune-facing WWP2 target described to date. In the SUSD6/TMEM127/WWP2 complex, WWP2 is recruited to MHC-I at membrane-associated compartments and promotes lysosomal degradation (28). This mechanism reduces tumor-cell visibility to CD8+ T cells. Unlike indirect associations between WWP2 and the microenvironment, this pathway provides a direct molecular explanation for how WWP2 can contribute to immune escape.

#### 5.1.3 DDX3X in diabetic vascular injury

In models of diabetic vascular injury, WWP2 has been associated with control of DDX3X and endothelial survival (17). Stress conditions that reduce WWP2 expression can increase endothelial apoptosis and vascular remodeling. This context is useful as a reminder that WWP2 activity may support tissue integrity in some settings. The therapeutic question is therefore not whether WWP2 is beneficial or harmful in general, but which substrate-specific function is active in the disease of interest.

#### 5.1.4 Notch proteins and tumor-suppressive WWP2 activity

Notch signaling provides a clear example of the potentially tumor-suppressive activity of WWP2. In breast cancer models, the long noncoding RNA BREA2 interferes with WWP2-mediated turnover of the Notch1 intracellular domain, thereby sustaining Notch signaling and metastatic behavior (22). A tumor-suppressive role has also been demonstrated in ovarian cancer, where WWP2 directly interacts with and ubiquitinates Notch3, attenuates Notch3 signaling, and suppresses tumor growth and Notch3-associated stem-like and platinum-resistant phenotypes (54). Notably, WWP2 loss or reduced expression was observed in a substantial proportion of ovarian carcinomas in that study (54). These findings contrast with the oncogenic effects of WWP2-mediated degradation of tumor suppressors such as PTEN or LATS1 and illustrate that the direction of WWP2 activity is determined by the biological function of the substrate being ubiquitinated. Thus, WWP2 should not be considered intrinsically oncogenic or tumor suppressive; rather, its net effect depends on the dominant substrate network within a particular tumor context.

#### 5.1.5 IRF7/Ccl5 in inflammation-fibrosis transition

WWP2 has been linked to inflammatory monocyte recruitment in cardiac fibrosis through an IRF7-Ccl5-associated pathway (15). This work places WWP2 at the interface of immune-cell trafficking and tissue remodeling. Although the evidence comes from cardiac disease rather than cancer, it provides a rationale for testing whether WWP2-dependent inflammatory recruitment contributes to fibrotic or immune-excluded tumor niches.

### 5.2 Physiological regulatory networks

#### 5.2.1 Ion transport and membrane protein homeostasis

WWP2 regulates selected membrane proteins through WW-domain-mediated recognition of PPxY motifs. A classical example is the epithelial sodium channel ENaC, for which WWP2 contributes to ubiquitin-dependent downregulation (55). Such activities show that WWP2 can control membrane-protein turnover beyond cancer and immunity, a feature relevant when considering potential on-target toxicities of WWP2-directed drugs.

#### 5.2.2 Transcriptional control and RNA processing

WWP2 has also been associated with regulation of RNA polymerase II and ADAR2 (56–58). These pathways connect WWP2 to transcriptional responses, DNA-damage-associated transcription arrest, stem cell biology, and RNA editing. Their relevance to cancer or immunity remains context dependent, but they broaden the range of processes that could be altered by global WWP2 inhibition.

#### 5.2.3 Signaling pathways relevant to cancer and immunity

PTEN-AKT and Hippo-YAP pathways illustrate how WWP2 can influence oncogenic state (18–20). TGF-beta-Smad and Notch signaling further show context dependence (12, 21, 22). Some of these pathways also shape immune phenotypes indirectly. For example, TGF-beta-associated programs can support T cell exclusion, and Hippo pathway changes can influence tumor immunity in selected models (43, 44). These connections are important background, but they should not be presented as direct evidence that WWP2 itself regulates every immune phenotype downstream of those pathways.

WWP2 target engagement is dynamic rather than fixed. Localization, WW-domain binding, HECT domain accessibility, and post-translational modification can all alter which substrate is modified (8–11). Isoform expression and adaptor abundance further shape context-dependent outcomes (12, 13). This explains why the same WWP2 protein may support PTEN turnover in one cancer model, regulate MHC-I trafficking in another, and participate in tissue-protective pathways elsewhere. Future studies should measure substrate occupancy and ubiquitination directly rather than inferring WWP2 function from expression alone.

### 5.3 Clinical evidence and translational questions

Available human evidence provides initial support for the clinical relevance of the SUSD6/TMEM127/WWP2 (STW) axis, although the evidence remains concentrated in a limited number of tumor types. In the study that defined this pathway, TCGA-based analyses showed correlated expression of SUSD6, TMEM127, and WWP2 in AML and representative solid tumors, including colorectal and hepatocellular carcinomas. The composite STW signature was elevated in AML and pancreatic adenocarcinoma (PAAD) relative to normal tissues, and higher STW signature levels were associated with poorer overall survival in both cohorts (28). In AML, higher SUSD6 expression was also associated with adverse clinical outcome and inversely correlated with a CD8+ T-cell activation signature in bone-marrow single-cell transcriptomic data (28). SUSD6 was additionally reported to be increased in several solid tumors, including PAAD, cholangiocarcinoma, glioblastoma, and lower-grade glioma, suggesting that enrichment of components of this pathway extends beyond AML.

However, important clinical gaps remain. The published TCGA analyses are primarily correlation- and signature-based and did not define a uniform expression threshold with which to estimate the fraction of individual tumors simultaneously exhibiting high SUSD6, TMEM127, and WWP2 expression. Moreover, direct clinical evidence linking WWP2 abundance or activity to tumor-cell surface MHC-I protein levels, spatial CD8+ T-cell infiltration, or response to immune-checkpoint blockade remains limited. This distinction is important because total WWP2 expression itself varies substantially among tumor types. A recent pan-cancer TCGA analysis, for example, reported increased WWP2 expression in LIHC, CHOL, PRAD, and STAD but decreased expression in several other cancers, including BRCA, COAD, KIRC, LUAD, and LUSC (59). Thus, total WWP2 transcript abundance cannot currently be regarded as a pan-cancer surrogate for activity of the STW complex. Current evidence instead supports AML and PAAD as tumor contexts with the strongest published cohort-level evidence for the STW signature, while validation in independent clinical cohorts, particularly those with matched MHC-I protein measurements, spatial immune profiling, and immunotherapy outcomes, remains an important priority.

The primary WWP2-dependent translational challenge lies in achieving target specificity. Broad catalytic inhibition could affect several WWP2 substrates at once and may therefore cause immune, vascular, or cardiac consequences (41, 42, 60). In cancer, the most tractable path may be to identify tumors that depend on the SUSD6/TMEM127/WWP2 pathway for MHC-I loss (28, 34, 35) and to disrupt the downstream MHC-I downregulation programs (36–39) selectively. Biomarkers should include WWP2 and adaptor expression, MHC-I abundance, antigen-presentation signatures, interferon signaling, CD8+ T cell infiltration, and response to immune checkpoint blockade (34–39).

A second priority is to define WWP2 function by cell type. Tumor cells, dendritic cells, macrophages, T cells, and fibroblasts may use different WWP2 substrates. Single-cell and spatial analyses, combined with ubiquitin proteomics and functional perturbation, will be needed to distinguish tumor-cell-intrinsic immune escape from immune-cell homeostatic functions. This information is necessary before a WWP2-targeted approach can be moved toward clinical testing.

## 6 Conclusion

WWP2 is a multifunctional HECT E3 ligase whose most immunologically important consequences are determined by substrate, adaptor, cell type, and disease context (5–13). In immune cells, WWP2 shapes B cell tolerance, helper T cell regulation, and macrophage stress responses (23–27). Related work on WWP2 regulation through adaptor systems and NDFIP proteins helps explain this context dependence (53, 61–63). In tumors, it can still influence proliferation, survival, and invasive behavior through pathways such as PTEN-AKT and LATS1-YAP (18–20). While important, these tumor-intrinsic mechanisms alone do not prove a role in immune escape.

WWP2 also regulates TRIF-dependent innate signaling and Itch-associated helper T cell biology (23–25). Independent work in B cells and macrophages further shows that WWP2 contributes to immune tolerance and host response outside the tumor compartment (26, 27).

A credible therapeutic strategy should therefore move beyond total WWP2 expression and focus on pathologic WWP2 complexes. Tumor-selective delivery and adaptor-selective disruption may offer a better balance between restoring immune surveillance and preserving normal WWP2 functions (28, 34, 35). Biomarker-guided patient selection should then focus on reduced MHC-I expression, antigen-presentation signatures, and broader immune-evasion programs (36–39). This is particularly relevant for tumors with reduced MHC-I expression or evidence of impaired antigen presentation.

In summary, WWP2 sits at the intersection of ubiquitin signaling, tumor-cell plasticity, and immune surveillance. Its biology is not adequately captured by calling it simply oncogenic or immunosuppressive. Defining when, where, and through which substrate WWP2 acts will be essential for turning a broad target network into precise therapeutic opportunities.
